# Follow-up after surgical treatment for intermittent claudication (FASTIC): a study protocol for a multicentre randomised controlled clinical trial

**DOI:** 10.1186/s12912-020-00437-7

**Published:** 2020-06-04

**Authors:** Sara Haile, Anneli Linné, Unn-Britt Johansson, Eva Joelsson-Alm

**Affiliations:** 1 Karolinska Institutet, Department of Clinical Science and Education, Södersjukhuset, and Department of Surgery, Södersjukhuset, Stockholm, Sweden; 2 Sophiahemmet University, Stockholm, Sweden; 3 Karolinska Institutet, Department of Clinical Science and Education, Södersjukhuset, and Department of Anaesthesiology and Intensive Care, Södersjukhuset, Stockholm, Sweden

**Keywords:** Intermittent claudication, Secondary prevention, Medication adherence, Vascular nursing, Person-centred care, Randomised controlled trial, atherosclerosis

## Abstract

**Background:**

Intermittent claudication (IC) is a classic symptom of peripheral arterial disease, and strongly associated with coronary heart disease and cerebrovascular disease. Treatment of IC and secondary prevention of vascular events include best medical treatment (BMT), changes in lifestyle, most importantly smoking cessation and increased physical exercise, and in appropriate cases surgery. A person-centred and health promotion approach might facilitate breaking barriers to lifestyle changes and increasing adherence to secondary prevention therapy. The FASTIC study aims to evaluate a nurse-led, person-centred, health-promoting follow-up programme compared with standard follow-up by a vascular surgeon after surgical treatment for IC.

**Methods:**

The FASTIC-study is a multicentre randomised controlled clinical trial. Patients will be recruited from two hospitals in Stockholm, Sweden after surgical treatment of IC through open and/or endovascular revascularisation and will be randomly assigned into two groups. The intervention group is offered a nurse-led, person-centred, health-promoting programme, which includes two telephone calls and three visits to a vascular nurse the first year after surgical treatment. The control group is offered standard care, which consists of a visit to a vascular surgeon 4–8 weeks after surgery and a visit to the outpatient clinic 1 year after surgical treatment. The primary outcome is adherence to BMT 1 year after surgical treatment and will be measured using The Swedish Prescribed Drug Registry. Clinical assessments, biomarkers, and questionnaires will be used to evaluate several secondary outcomes, such as predicted 10-year risk of cardiovascular and cerebrovascular events, health-related quality of life, and patients’ perceptions of care quality.

**Discussion:**

The FASTIC study will provide important information about interventions aimed at improving adherence to medication, which is an unexplored field among patients with IC. The study will also contribute to knowledge on how to implement person-centred care in a clinical context.

**Trial registration:**

ClinicalTrials.govNCT03283358, registration date 06/13/2016.

## Introduction

Intermittent claudication (IC) is a classic symptom of peripheral arterial disease (PAD), which is caused by atherosclerosis, and leads to decreased arterial circulation to the lower extremities. Clinical symptoms of PAD vary from atypical claudication with inconsistent leg discomfort on effort, IC with pain induced by exercise, to critical limb ischaemia (CLI) with ischaemic rest pain and ulceration or gangrene. Age, heredity, lifestyle (smoking, diet, and lack of exercise), high blood pressure, and diabetes are some of the risk factors that contribute to formation of atherosclerosis [1, 2]. IC is strongly associated with coronary heart disease and cerebrovascular disease. Symptoms of atherosclerotic disease from the lower extremities can be an early predictor of all-cause and cardiovascular-related mortality [3].

IC affects approximately 7% of all adult individuals older than 60 years [4]. PAD results in chronic pain, impaired walking ability, restricted mobility, and reduced ability to perform daily activities. People suffering from IC are burdened by social isolation, fatigue, and a sense of dependency, resulting in decreased quality of life [5, 6].

Treatment of IC aims at symptom relief, increased walking ability, increased health-related quality of life (HRQoL), and secondary prevention of vascular events. Management of IC according to clinical guidelines, include best medical treatment (BMT), changes in lifestyle, most importantly smoking cessation and increased physical exercise, and in appropriate cases, revascularisation through surgical or endovascular methods [1, 2, 7]. An increased treadmill walking capacity can be achieved when endovascular therapy is added to medical therapy or supervised exercise during early and intermediate follow-up [8]. Fahkry et al. showed that combination therapy with endovascular revascularisation in addition to supervised exercise provides superior improvement in walking distance and HRQoL compared with supervised exercise alone [9].

Regardless of the need for revascularisation, those diagnosed with IC should be treated with BMT to prevent progression of disease and reduce the risk for cardiovascular and cerebrovascular complications and death [7]. BMT for patients with IC includes antiplatelet agents, lipid-lowering agents, and antihypertensive treatment in the presence of hypertension [1, 10]. A large meta-analysis showed a 23% reduction in risk, using antiplatelet agents, of serious cardiovascular events in 9214 patients with PAD [11]. An observational, retrospective cohort study that analysed data from the Swedish national health care registries showed that only 65% of the patients with IC were offered BMT, which was defined as any antiplatelet or anticoagulant therapy along with statin treatment [12].

### Scientific background

Complex interventions are widely used in the health service and defined as interventions.

which comprise multiple interacting component [13]. Medical Research Council (MRC) guidelines for developing and evaluating complex interventions provide guidance to researchers and strengthen the internal and external validity and add value to health care research. Several steps are recommended for the systematic development of complex interventions such as problem identification, the systematic identification of evidence, identification or development of theory, determination of needs, the examination of current practice and context, modelling the process and expected outcomes leading to final element: the intervention design [13, 14].

Person-centred care (PCC) is a method of care-giving that has been implemented over the last few years. It has been shown to improve self-efficacy, adherence to prescribed medication and shorten hospital stay [15]. Person-centred care (PCC) means involvement of the patients as partners in their care. The core of a person-centred approach is allowing the patient to be part of their care by letting them describe their situation from their own point of view and putting their views at the centre of care. This could also be described as a collaborative process of decision-making between patients and their health care provider that takes into account the patient’s values and preferences and clinical evidence [16]. A personal health plan is made based on the information exchanged during the narrative communication. This health plan includes goals, strategies, and terms of evaluation, and is jointly developed by the patient and the professional [17]. The process of practicing PCC in health care as described by Ekman et al. contains three components: patient narratives, partnership, and safeguarding the partnership [17]. Patient narratives is about sharing of information and initiating partnership between the patient and the care giver. Narrative communication involves learning from each other, creating common understanding of the illness, and learning about the patient’s resources, limitations, and expectations. This provides the professional a good basis for discussing and planning care and treatment with the patient [17]. A person-centred care approach added to the usual care of patients with acute coronary syndrome improves general self-efficacy, which is an important factor for effective self-management of illness [18].

Effective public health, health promotion, and chronic disease management programmes help people maintain and improve health, reduce disease risks, and manage chronic illness. Health behaviour theory can play a critical role throughout the programme planning process and for the effect of the intervention. According to social cognition theory, self-efficacy, goals, and outcome expectancies are three main factors that can affect the possibility that a person will change a health behaviour are [19].

Secondary prevention therapy requires engagement, participation, and adherence from the patient. Patients with PAD require disease-specific information, adequate support enabling them to manage recommended behavioural changes [20] and to adapt to and live with chronic illness [21]. A large international registry on patients with established atherosclerosis reported an association between non-adherence to evidence-based secondary prevention therapies and a significant increase in risk for long-term adverse events, including mortality [22]. Adherence to medication has been reported to be as low as 30% [23] to 50% [22]. Non-adherence to medication after acute coronary syndrome tends to be lower immediately after discharge (20%) and increases to 54 and 53% at 6 months and 1 year after discharge, respectively [24]. Barriers to physical activity in patients with PAD are related to the pain experienced when walking [25], lack of knowledge about the condition and doubts that lifestyle changes, particularly physical activity, would improve their condition [20]. Therefore, providing clear and consistent information about the condition and detailed information regarding exercise, as well as mental support to enable behavioural changes, are important [20]. Furthermore, preventive treatment measures require a long-term follow up. A 20-min visit with a vascular surgeon in the standard follow-up after surgical intervention offers limited time to address the important questions of physical exercise, smoking, and medical adherence. Probably not all the patient’s barriers to lifestyle changes and medical adherence are identified in such a visit. A person-centred and health promotion approach might facilitate breaking barriers and increasing adherence to secondary prevention therapy for patients with IC. However, to the best of our knowledge, no such studies have been performed.

### Hypothesis

We hypothesise that a nurse-led, person-centred, health-promoting follow-up programme will increase adherence to BMT and reduce the risk for cardiovascular or cerebrovascular events compared with standard care.

## Methods/design

### Objectives

The overall aim of the FASTIC study is to evaluate a nurse-led, person-centred, health-promoting follow-up programme compared with standard follow-up by a vascular surgeon after surgical treatment for IC.

The primary objective of the FASTIC study is to examine the effect of the above-mentioned programme on the patient’s adherence to prescribed BMT (primary outcome) and reduce the risk for cardiovascular or cerebrovascular events. Furthermore, secondary objectives of the FASTIC study are to investigate the effect of the above-mentioned programme on patient-reported outcomes (HRQoL, health literacy, belief of medication, and adherence to medication), health outcomes, and participants’ experience of the intervention programme compared with the standard follow-up.

### Study design and setting

The FASTIC study is a multicentre, randomised controlled clinical trial. Patients will be recruited from two sites; the Departments of Vascular Surgery at Södersjukhuset and at Karolinska University Hospital, both in Stockholm, Sweden. Patients with IC are referred to the vascular surgical outpatient clinics in one of these hospitals by a primary care physician.

The Stockholm County is a geographical area covering the City of Stockholm and surrounding area with approximately 2.3 million inhabitants. This region has two centres for vascular surgery, the Karolinska University Hospital and Södersjukhuset, both of which are located near the city centre. On average, 350 open surgical interventions and endovascular interventions for patients with IC are performed at these two centres every year according to the Swedish National Registry for Vascular Surgery (Swedvasc). No major vascular surgery is performed outside these centres and all patients in the Stockholm County with IC who may require arterial surgical intervention are referred to one of these centres. During the first visit at the vascular surgical outpatient clinic, diagnosis and requirement for revascularisation are decided by a vascular surgeon and recorded in the patient’s medical record. This decision is based on clinical symptoms and circulatory assessments, including the ankle brachial index (ABI), Duplex ultrasound, and/or computed tomography angiography. Cessation of smoking for at least 3 months prior to surgery is required. Patients are prescribed statins and antiplatelet agents at this point if not previously prescribed.

### Eligibility criteria

Patients who fulfil all the inclusion criteria and none of the exclusion criteria mentioned below are eligible for the study.

#### Inclusion criteria

- Age of 18 years and older
- Diagnosed with IC (ICD-10 Diagnosis Code I70.2 or I739B) by a vascular surgeon
- No symptoms or signs of critical limb ischaemia (rest pain, ulceration, or gangrene)
- Scheduled for revascularisation through open and/or endovascular surgery
- Ability to speak and understand Swedish language

#### Exclusion criteria

- Diagnosed with dementia
- Planned discharge to a nursery home
- Not accountable for administrating their own medications
- A survival expectancy less than 1 year

### Sample size

The sample size was calculated based on results from previous literature regarding primary outcome (adherence to medication) in cardiological studies [22, 26]. Statistical power analysis using IBM SPSS Sample Power 3 (IBM Corp., Armonk, NY, USA) showed that a required sample size of 186 participants was needed to detect a statistically significant increase in adherence to prescribed BMT from 50 to 70% (power, 0.80; significance value, 0.05; two-sided). We plan to recruit 210 participants and expect a 10% drop-out.

### Recruitment

**Fig. 1:**
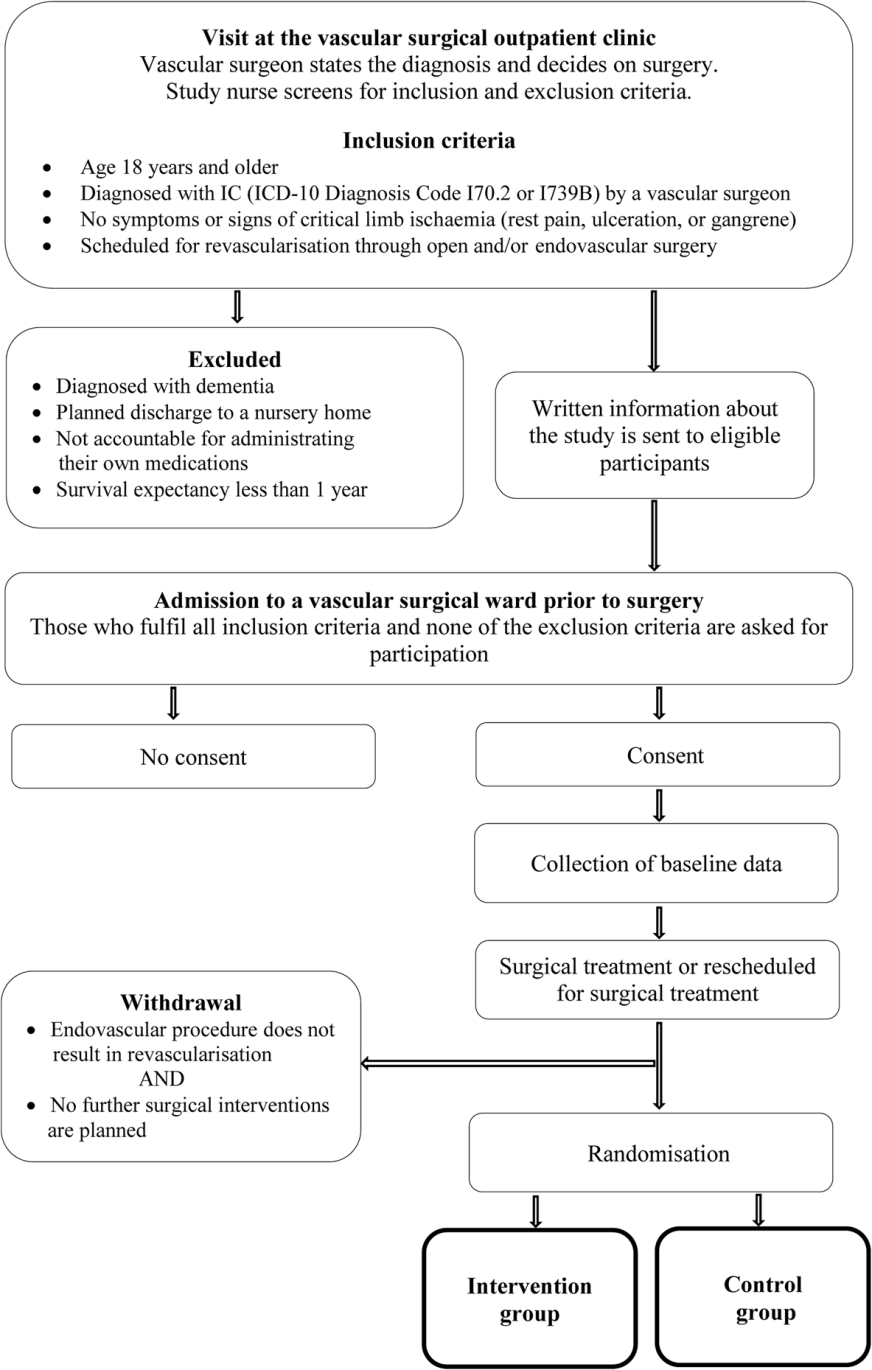
Study flow diagram of enrolment and randomisation

**Fig. 2:**
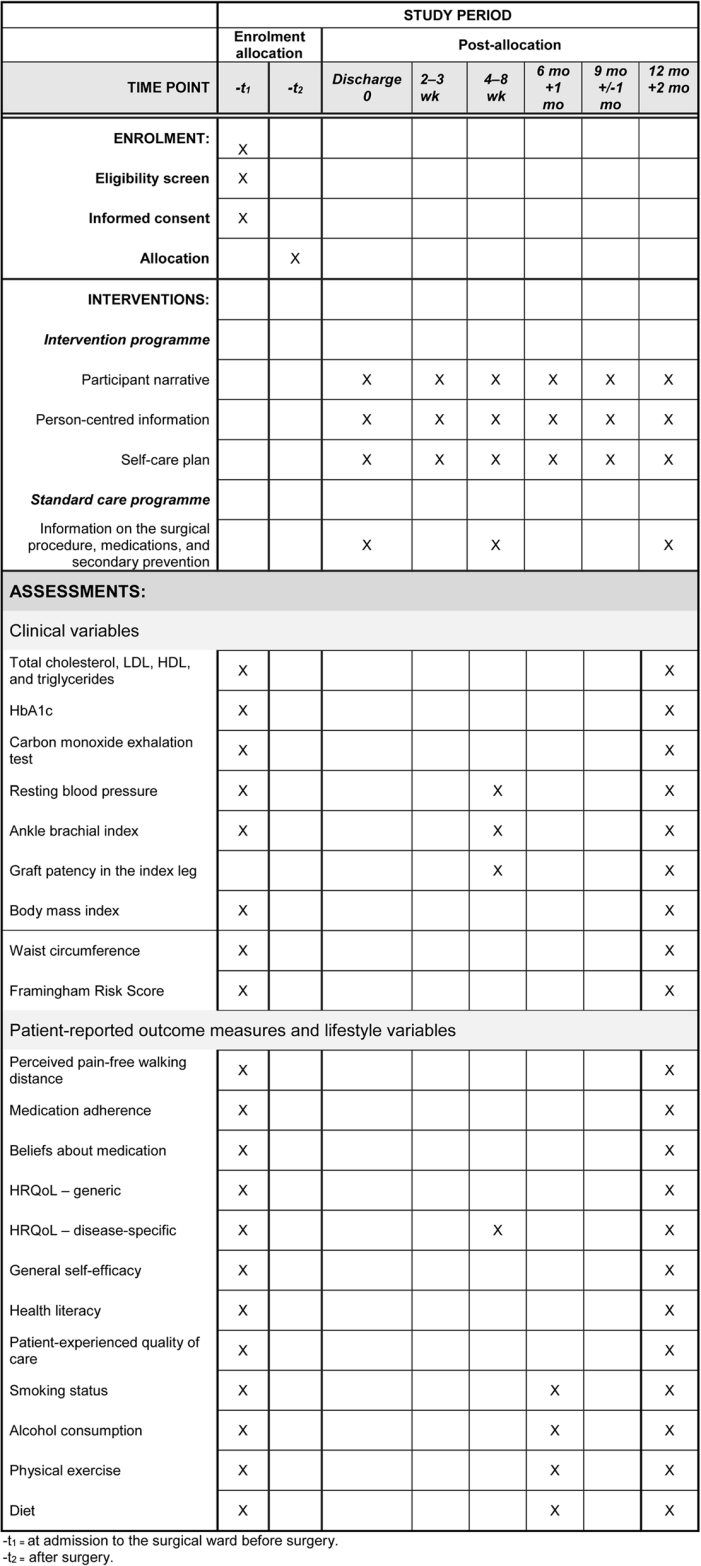
SPIRIT schedule of enrolment, interventions, and assessments

Patients who are eligible for the study are identified by one of the study nurses at each of the two outpatient clinics. Written information about the FASTIC study, including a consent form, is sent by mail to the eligible patients. When the patients arrive to the vascular surgical ward prior to surgery, the study nurse provides oral information about the study and asks for participation. If consent is obtained, baseline data are collected. The surgery is performed and randomisation to either an intervention programme (intervention group) or a standard care programme (control group) is performed immediately after surgery by a study nurse. In some cases, the endovascular procedure may not result in revascularisation. The patient can still be randomised to the FASTIC study if a decision is made to reschedule to open surgery. If no further surgical interventions are planned, the patient will be withdrawn from the study. Figure 1 shows a description of the study flow. A time schedule of study enrolment, interventions, and assessments is shown in Fig. 2.

### Randomisation

Randomisation is performed by sequence generation using secure computer-generated random numbers. The allocating study nurses have no insight to block randomisation factors behind the sequence generation process. Patients who meet all study criteria and consent to participate are randomised to either the intervention group or the control group. No blinding will be applied in this study.

### Interventions

#### Standard care programme

**Fig. 3:**
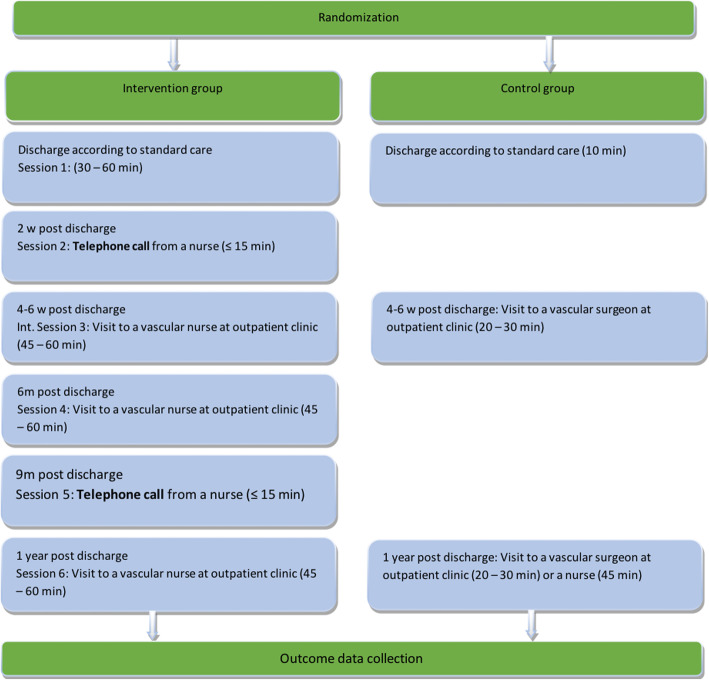
Description of outpatient visits and telephone calls with estimated time required for participants in the intervention group vs the control group

Participants in the standard care programme (control group) will receive written and verbal information about their surgical procedure, described medications, and secondary prevention during the hospital stay at the surgical ward. The standard follow-up after discharge consists of two visits at the outpatient clinic (Fig. 3):

- Visit 1: 4–8 weeks after revascularisation. The participant meets a vascular surgeon during 20 min.
- Visit 2: 1 year after revascularisation. The majority of the participants meet a vascular nurse during 30–40 min. Some participants with more complicated cases will meet a vascular surgeon during 20 min instead of a nurse, and this decision is made during visit 1.

During these visits, the participants are managed following guideline-directed care^2^, which includes interval history (new symptoms) and vascular examination of the leg and the surgical site. A pharmaceutical review of the participant’s medical treatment is performed, and information of secondary preventive measures is supposed to be provided.

#### Intervention programme

Participants in the intervention programme (intervention group) are also managed following guideline-directed care [2], but will also receive a nurse-led, person-centred, health-promoting follow-up programme in accordance with the PCC approach described by Ekman et al. [17]. The vascular nurses in this programme (FASTIC nurses) are specially trained in PCC and in motivational interviewing (MI). MI is a method focused on helping patients adhere to treatment recommendations by exploring their personal perspectives and ambivalence, identifying barriers, and promoting behavioural changes [27]. A partnership between the participant and the FASTIC nurse is initiated at a meeting at the surgical ward before discharge. The following programme consists of three visits at the outpatient clinic and two telephone calls (Fig. 3). All contacts between the participant and the FASTIC nurse are based on the participant’s own narratives and a joint agreement on a self-care plan. The intervention programme includes some questions and reflections about the participant’s lifestyle. That could be experienced as intrusion by some participants. Therefore, all conducted dialogs will be based on the individual patient’s needs and wishes. The nurses will have regular meetings with the principal investigator to discuss methodology, approaches, and any issues that arise in the process.

##### **Participant narrative** (Fig. 4)

In a narrative, the participant describes their self-perceived health history, previous knowledge on the disease, risk factors, received treatment, and expectations of the future. Furthermore, the participant recounts their capabilities and limitations to meet their own self-care goals (e.g., behaviour/lifestyle changes and adherence to medications.

**Fig. 4:**
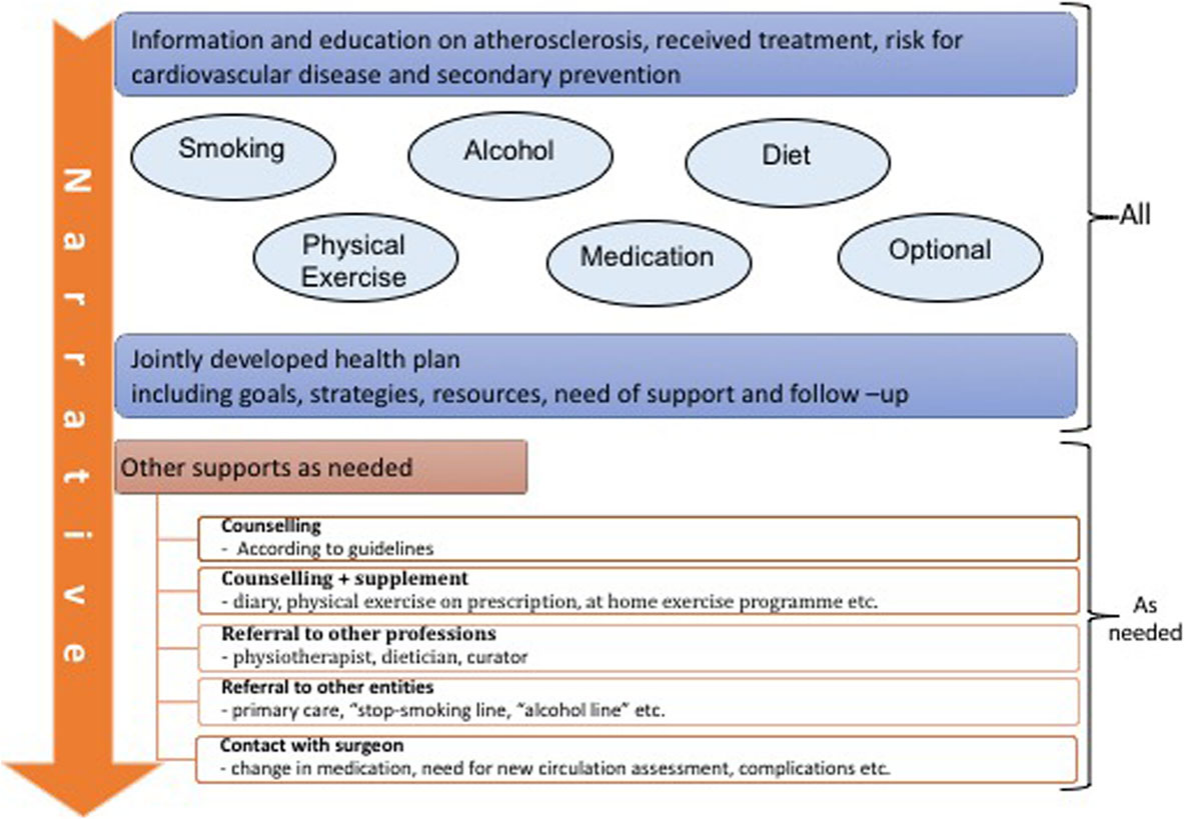
The main content of the participants’ narrative

Based on the narrative, the FASTIC nurse and the participant discusses relevant information on the disease (arthrosclerosis), received surgical treatment, the increased risk for future cardiovascular and cerebrovascular events due to cardiovascular disease, available BMT, and modification of risk factors (smoking cessation, healthy diet, physical activity, and adherence to medication). The information from the nurse is provided by dialog using open-ended questions, reflective listening, and summary statements inspired by MI. Different methods (printed material, schematic pictures, and short films) are used if the participant needs specific information. Individualised advice and recommendations on lifestyle (diet, alcohol consumption, and physical exercise) are based on the European guidelines on cardiovascular disease prevention in clinical practice [28], the Swedish national guidelines for disease prevention methods [11], the Swedish national guidelines for physical activity in the prevention and treatment of disease [29] and Nordic nutrition recommendations [30].

##### Self-care plan

After summarising the narrative and the dialog described above, a joint agreement on a self-care plan is established and documented in the medical record. The self-care plan contains a detailed description on goals, strategies, the participant’s own capabilities to achieve the goals and the need of support from the FASTIC nurse. Examples of such support are referrals to expertise (e.g., for smoking cessation, physical therapy, contact with dieticians) or to primary care. The FASTIC nurse provides strategies to improve adherence to medications and to lifestyle changes. The written agreement of the self-care plan is provided to the participant either before discharge or sent by post within 1 week of discussion during the information session. During every contact between the participant and the FASTIC nurse, the self-care plan is reviewed and modified if required.

Because each participant has his/her own personalised self-care plan, there are no mandatory interventions. All interventions will be documented in the participant’s case report form and reported at the end of the study.

### Data collection

#### Biomarkers

Biomarkers will be measured at study enrolment and at 1 year after revascularisation. Blood samples will be collected to assess HbA1c, triglyceride, high- and low-density cholesterol, and total cholesterol levels. An exhalation test for measuring carbon monoxide, the piCO™ Smokerlyzer® (Bedfont® Scientific Ltd., Kent, England), will be used to assess smoking status.

#### Adherence to medication

The primary outcome, adherence to medication, is defined as adherence to prescribed antithrombotic agents and lipid modifying agents 1 year after revascularisation.

The Swedish Prescribed Drug Registry (PDR) will be used for collecting data on medication adherence. The PDR provides data on prescribed drugs containing information on dispensed items, dispensed amount, dosage, and expenditure. Furthermore, this registry has been linked to the civic registry number since 2005 and thus provides information on an individual basis. This registry does not include medications that are prescribed and administered in hospitals [31]. Data from a period of 18 months (6 months before to 12 months after revascularisation) will be retrieved from the PDR for each participant. The WHO Collaborating Centre for Drug Statistics Methodology classification of Anatomical Therapeutic Chemical (ATC) codes will be used to identify antithrombotic agents and lipid-modifying agents (see Table 1 for specific codes). Although antihypertensive medications will not be included in the analysis for primary outcome, they can be considered as BMT for patients with hypertension. Information on antihypertensives will be retrieved from the PDR as medication of interest for the study. We will also collect patient-reported adherence to medication (anticoagulant agents, antiplatelet agents and lipid-lowering agents) at study enrolment and at 1 year after revascularisation.

**Table 1:** Medications of interest in the study

Medication group	ATC Code
Antithrombotic agents	
Vitamin K antagonists	B01AA
Heparin group	B01AB
Platelet aggregation inhibitors excluding heparin	B01AC
Direct thrombin inhibitors	B01AE
Direct factor Xa inhibitors	B01AF
Lipid-modifying agents	
HMG CoA reductase inhibitors	C10AA
Fibrates	C10AB
Other lipid-modifying agents	C10AX
Antihypertensives	
Diuretics	C03
Beta-blocking agents	C07
Calcium channel blockers	C08
ACE inhibitors, plain	C09A
ACE inhibitors, combinations	C09B
Angiotensin II antagonists, plain	C09C
Angiotensin II antagonists, combinations	C09D

legend: Medications of interest in the study and their Anatomical Therapeutic Chemical (ATC) codes according to the WHO Collaborating Centre for Drug Statistics Methodology (WHOCC) classification*.*

### Assessment of long-term risk for cardiovascular events

The Framingham Risk Score (FRS) [32] will be used to calculate the predicted 10-year risk of cardiovascular and cerebrovascular events. FRS sex-specific prediction formulas are based on general cardiovascular risk factors (age, systolic blood pressure, treatment for hypertension, smoking status, diabetes, and total and high-density cholesterol levels). The FRS will be combined with the ABI, which have been shown to improve the accuracy of risk prediction [33].

### Clinical assessments

Resting blood pressure, weight, height, calculation of body mass index (BMI), and waist circumference will be measured at study enrolment and at 1 year after revascularisation. The patients will also be asked to state their perceived pain-free walking distance.

ABI, defined as the ratio of systolic blood pressure at the ankle and systolic blood pressure in the upper arm, will be measured and calculated at study enrolment, after 4–8 weeks, and at 1 year after revascularisation.

Graft patency will be assessed using the ABI and the patients’ history. If there are any indications of re-occlusion, a Duplex ultrasound examination will be performed.

### Questionnaires

*Beliefs about medication* (BMQ) will be measured by the BMQ questionnaire [34]. This questionnaire consists of two parts, which are the BMQ-General and BMQ-Specific. The BMQ-General comprises three four-item scales assessing general beliefs about pharmaceuticals as a form of treatment. The General Harm scale assesses beliefs about the degree to which medicines are essentially harmful. The General Overuse scale assesses beliefs about whether doctors place too much emphasis and trust on medicines. The General Benefit scale assesses beliefs about the degree to which medicines are fundamentally beneficial. The BMQ-Specific comprises two scales assessing beliefs about the necessity of a specific treatment for controlling an illness (5 items) and concerns about potential adverse consequences of taking it (6 items). Each item has a five-point response scale (1 = strongly disagree to 5 = strongly agree). The validity of this questionnaire has previously been confirmed [34, 35]. Agreement to use the BMQ questionnaire has been obtained from Professor Rob Horne.

*Health literacy* (HL) will be measured using the Swedish version of the European Health Literacy questionnaire, HLS-EU-Q16 [36]. The short version of this questionnaire consists of 16 items. These items focus on four HL dimensions reflecting perceived ease or difficulty in an individual’s ability to accessing/obtaining health information, understanding health information (not only in written form), processing/appraising health information, and applying/using health information. Response choices are “very easy”, “easy”, “difficult”, and “very difficult” [36].

*HRQoL* will be measured using the Swedish versions of EQ-5D 5 L [37, 38] and the Vascular Quality of Life Questionnaire (VascuQol-6) [39]. The EQ-5D 5 L is a generic instrument from the EuroQol Group [40] and measures health in five dimensions (mobility, self-care, usual activities, pain/discomfort, and anxiety/depression). Each dimension has five levels of severity (no problems, slight problems, moderate problems, severe problems, and extreme problems). This questionnaire includes EQ-VAS, where the participant grades their own perceived health status on a visual analogue thermometer scale with endpoints of 0–100 (0 = the worst health you can imagine and 100 = the best health you can imagine) [37]. VascuQoL-6 is a disease-specific instrument and consists of six items (symptom, pain, social life, emotional, and two items in activity) and each item has a four-point response scale [39]. Previous studies have shown that VascuQol-6 is a valid and reliable instrument for assessing HRQoL in patients with PAD [41].

The *General Self Efficacy* (GSE) scale measures people’s beliefs in their abilities to cope with a variety of difficult demands in life. The GSE scale consists of 10 items rated on a four-point Likert scale (1 = not at all true, 2 = hardly true, 3 = moderately true, and 4 = exactly true), yielding a total score between 10 and 40. The GSE scale was originally developed in Germany [42] and has been validated in Swedish [43].

*Patient-experienced quality of care* will be measured by using the questionnaire Quality from the Patient’s Perspective (QPP) [44]. In QPP, the items are evaluated by the respondent in two ways. To measure the subjective importance that the person ascribes to the various aspects of care, each item is related to the statement “This is how important it was to me …” . A four-point response scale ranging from 1 (of little or no importance) to 4 (of the very highest importance) is used for all items. To measure the perceived reality of the quality of care, each item is related to the sentence “This is what I experienced …” . In this part of QPP, a four-point response scale ranging from 1 (do not agree at all) to 4 (completely agree) is used. The short version of QPP will be used in the study [45]. The validity of QPP has been tested previously [44, 45].

*A screening instrument of unhealthy lifestyle habits* from the Swedish National Board of Health and Welfare’s National Guidelines for Methods of Preventing Disease will be used when establishing the self-care plan with the participants in the intervention group and during the follow-up sessions. This instrument includes items on tobacco use, alcohol intake, physical activity, and eating habits [11].

### Data management

All electronic study data will be stored in a password-protected local database and participants will only be identifiable by their study number. All paperwork will be kept in a locked filing cabinet in a locked office.

### Statistical analysis

Descriptive statistics will be used to describe baseline demographic and clinical data. Continuous variables will be summarised as mean (SD) or median (interquartile range), and categorical variables will be summarised as number (percentage). Comparisons between the intervention group and the control group will be made using the t-test and ANOVA for repeated measures, or the Wilcoxon rank-signed test and Kruskall–Wallis test according to the underlying distribution for continuous data. The chi-square or Fischer’s exact test will be used for categorical data. Logistic regression analysis will be performed for binary outcomes to adjust for confounding factors. All analyses will be performed according to the intention-to-treat principle. IBM SPSS version 23 or later (IBM Corp., Armonk, NY, USA) will be used for statistical analyses. The CONSORT 2010 statement will be followed when reporting the results [46].

### SPIRIT statement

The protocol has been designed according to SPIRIT Recommendations of the Standard Protocol Items: Recommendations for Interventional Trials [47]. A SPIRIT figure is available for this protocol (Fig. 2).

### Trial status

The FASTIC trial is currently ongoing with data collection in both study sites. Recruitment of participants started in June 2016 and was completed in November 2018.

## Discussion

The global aim of our research project is to reduce cardiovascular-related morbidity and mortality, which is currently one of the largest public health problems. In the FASTIC study, we will target patients with IC, which is a cohort with high risk of future cardiovascular and cerebrovascular events. Furthermore, most patients with IC are of older age and a recent review stated that there is a knowledge gap on optimal strategies for primary and secondary prevention of cardiovascular disease among older adults (> 75 years) [48]. The authors also concluded that there is a need for clinical trials using novel study designs with person-centred outcomes.

The concept of person-centred care has been highlighted recent years, and there is an increasing demand from national authorities to improve the quality of care by adapting health care to a more person-centred model. The FASTIC study can contribute to knowledge on how to implement person-centred care in a clinical context. We also believe that the results from our study can provide important information about interventions aimed to improve adherence to medication, which is an unexplored field among patients with IC, and other secondary prevention and risk reduction therapies.

The clinical context of the FASTIC study can be both a strength and a limitation. The methods used and evaluated in this study can be easily implemented in other clinical settings. However, uncontrolled conditions and events that may occur in a clinical context can affect the reliability of the results. The diversity of interventions in the intervention programme may also appear to be a reliability problem, but this is an inevitable consequence of personalised care that is fundamental in person-centred care.

Attrition, loss of participants during the intervention could be a potential threat to the internal and external validity in RCTs. The expected drop-out rate for this RCT is 10% and is taken into account in the sample size calculation. Furthermore, to reduce the risk of response burden we have based decision on use of questionnaire on the content rather than the length per se which is also described as preferable [49]. Process evaluation within studies can be used to assess fidelity and quality of implementation, clarify causal mechanisms and identify contextual factors associated with variation in outcomes [50]. Attendance and notes on reasons for absence at individual visits will be recorded continuously. The intervention is theory driven and the nurses are specially trained in PCC approach and in MI. A qualitative interview study is planned with the aim of exploring the participants‘experiences after attending 1 year of the FASTIC study.

## Data Availability

The datasets used and/or analysed during the current study will be available from the corresponding author on reasonable request. Results of this trial will be presented at national and international conferences, and published in relevant medical peer-reviewed journals.

## Abbreviations

IC: Intermittent claudication
BMT: Best medical treatment
FASTIC: Follow-up after surgical treatment for intermittent claudication
PAD: Peripheral arterial disease
CLI: Critical limb ischaemia
HRQoL: Health-related quality of life
PCC: Person-centred care
MI: Motivational interviewing
PDR: Swedish prescribed drug registry
ATC: Anatomical therapeutic chemical
FRS: The Framingham risk score
ABI: Ankle brachial index
Swedvasc: Swedish national registry for vascular surgery
BMI: Body mass index
HL: Health literacy

## References

[1] Kiernan TJ, Hynes BG, Ruggiero NJ, Yan BP, Jaff MR (2010). Comprehensive evaluation and medical management of infrainguinal peripheral artery disease: "when to treat, when not to treat". Tech Vasc Interv Radiol.

[2] Norgren L, Hiatt WR, Dormandy JA, Nehler MR, Harris KA, Fowkes FG (2007). Inter-Society Consensus for the Management of Peripheral Arterial Disease (TASC II). J Vasc Surg.

[3] Newman AB, Shemanski L, Manolio TA, Cushman M, Mittelmark M, Polak JF, Powe NR, Siscovick D (1999). Ankle-arm index as a predictor of cardiovascular disease and mortality in the cardiovascular health study. The cardiovascular health study group. Arterioscler Thromb Vasc Biol.

[4] Sigvant B, Wiberg-Hedman K, Bergqvist D, Rolandsson O, Andersson B, Persson E, Wahlberg E (2007). A population-based study of peripheral arterial disease prevalence with special focus on critical limb ischemia and sex differences. J Vasc Surg.

[5] Egberg L, Andreassen S, Mattiasson AC (2012). Experiences of living with intermittent claudication. J Vasc Nurs.

[6] Wann-Hansson C, Hallberg IR, Klevsgard R, Andersson E (2005). Patients' experiences of living with peripheral arterial disease awaiting intervention: a qualitative study. Int J Nurs Stud.

[7] Aboyans V, Ricco JB, MEL B, Bjorck M, Brodmann M, Cohnert T, Collet JP, Czerny M, De Carlo M, Debus S (2018). 2017 ESC Guidelines on the Diagnosis and Treatment of Peripheral Arterial Diseases, in collaboration with the European Society for Vascular Surgery (ESVS): Document covering atherosclerotic disease of extracranial carotid and vertebral, mesenteric, renal, upper and lower extremity arteriesEndorsed by: the European Stroke Organization (ESO) The Task Force for the Diagnosis and Treatment of Peripheral Arterial Diseases of the European Society of Cardiology (ESC) and of the European Society for Vascular Surgery (ESVS). Eur Heart J.

[8] Ahimastos AA, Walker PJ, Askew C, Leicht A, Pappas E, Blombery P, Reid CM, Golledge J, Kingwell BA (2013). Effect of ramipril on walking times and quality of life among patients with peripheral artery disease and intermittent claudication: a randomized controlled trial. JAMA..

[9] Fakhry F, Spronk S, van der Laan L, Wever JJ, Teijink JA, Hoffmann WH, Smits TM, van Brussel JP, Stultiens GN, Derom A (2015). Endovascular revascularization and supervised exercise for peripheral artery disease and intermittent claudication: a randomized clinical trial. JAMA..

[10] Pennywell DJ, Tan TW, Zhang WW (2014). Optimal management of infrainguinal arterial occlusive disease. Vasc Health Risk Manag.

[11] Antithrombotic Trialists C (2002). Collaborative meta-analysis of randomised trials of antiplatelet therapy for prevention of death, myocardial infarction, and stroke in high risk patients. BMJ..

[12] Sigvant B, Kragsterman B, Falkenberg M, Hasvold P, Johansson S, Thuresson M, Nordanstig J (2016). Contemporary cardiovascular risk and secondary preventive drug treatment patterns in peripheral artery disease patients undergoing revascularization. J Vasc Surg.

[13] Craig P, Dieppe P, Macintyre S, Michie S, Nazareth I, Petticrew M (2013). Developing and evaluating complex interventions: the new Medical Research Council guidance. Int J Nurs Stud.

[14] Bleijenberg N, de Man-van Ginkel JM, Trappenburg JCA, Ettema RGA, Sino CG, Heim N, Hafsteindottir TB, Richards DA, Schuurmans MJ (2018). Increasing value and reducing waste by optimizing the development of complex interventions: enriching the development phase of the Medical Research Council (MRC) framework. Int J Nurs Stud.

[15] Ekman I, Wolf A, Olsson LE, Taft C, Dudas K, Schaufelberger M, Swedberg K (2012). Effects of person-centred care in patients with chronic heart failure: the PCC-HF study. Eur Heart J.

[16] Makoul G, Clayman ML (2006). An integrative model of shared decision making in medical encounters. Patient Educ Couns.

[17] Ekman I, Swedberg K, Taft C, Lindseth A, Norberg A, Brink E, Carlsson J, Dahlin-Ivanoff S, Johansson IL, Kjellgren K (2011). Person-centered care--ready for prime time. Eur J Cardiovasc Nurs.

[18] Fors A, Ekman I, Taft C, Bjorkelund C, Frid K, Larsson ME, Thorn J, Ulin K, Wolf A, Swedberg K (2015). Person-centred care after acute coronary syndrome, from hospital to primary care - a randomised controlled trial. Int J Cardiol.

[19] National Cancer Institute (2012). U.S. Department of Health and Human Services, National Institutes of Health: Theory at a Glance: A Guide for Health Promotion Practice.

[20] Gorely T, Crank H, Humphreys L, Nawaz S, Tew GA (2015). "standing still in the street": experiences, knowledge and beliefs of patients with intermittent claudication--a qualitative study. J Vasc Nurs.

[21] Wann-Hansson C, Rahm Hallberg I, Klevsgard R, Andersson E (2008). The long-term experience of living with peripheral arterial disease and the recovery following revascularisation: a qualitative study. Int J Nurs Stud.

[22] Kumbhani DJ, Steg PG, Cannon CP, Eagle KA, Smith SC, Hoffman E, Goto S, Ohman EM, Bhatt DL (2013). Adherence to secondary prevention medications and four-year outcomes in outpatients with atherosclerosis. Am J Med.

[23] Granger BB, Ekman I, Granger CB, Ostergren J, Olofsson B, Michelson E, McMurray JJ, Yusuf S, Pfeffer MA, Swedberg K (2009). Adherence to medication according to sex and age in the CHARM programme. Eur J Heart Fail.

[24] Kalichman SC, Kalichman MO, Cherry C, Hoyt G, Washington C, Grebler T, Merely C, Welles B (2015). Intentional medication non-adherence due to interactive toxicity beliefs among HIV positive active drug users. J Acquir Immune Defic Syndr.

[25] Barbosa JP, Farah BQ, Chehuen M, Cucato GG, Farias Junior JC, Wolosker N, Forjaz CL, Gardner AW, Ritti-Dias RM (2015). Barriers to physical activity in patients with intermittent claudication. Int J Behav Med.

[26] Granger BB, Ekman I, Hernandez AF, Sawyer T, Bowers MT, DeWald TA, Zhao Y, Levy J, Bosworth HB (2015). Results of the chronic heart failure intervention to improve MEdication adherence study: a randomized intervention in high-risk patients. Am Heart J.

[27] Levensky ER, Forcehimes A, O'Donohue WT, Beitz K (2007). Motivational interviewing: an evidence-based approach to counseling helps patients follow treatment recommendations. Am J Nurs.

[28] Piepoli MF, Hoes AW, Agewall S, Albus C, Brotons C, Catapano AL, Cooney MT, Corra U, Cosyns B, Deaton C (2016). 2016 European Guidelines on cardiovascular disease prevention in clinical practice: The Sixth Joint Task Force of the European Society of Cardiology and Other Societies on Cardiovascular Disease Prevention in Clinical Practice (constituted by representatives of 10 societies and by invited experts). Developed with the special contribution of the European Association for Cardiovascular Prevention & Rehabilitation (EACPR). Eur Heart J.

[29] Swedish National Institute of Public Health (2010). Physical Activity in the Prevention and Treatment of Disease.

[30] Nordic Council of Ministers (2012). Nordic Nutrition Recommendations.

[31] Wettermark B, Hammar N, Fored CM, Leimanis A, Otterblad Olausson P, Bergman U, Persson I, Sundstrom A, Westerholm B, Rosen M (2007). The new Swedish prescribed drug register--opportunities for pharmacoepidemiological research and experience from the first six months. Pharmacoepidemiol Drug Saf.

[32] Wilson PW, D'Agostino RB, Levy D, Belanger AM, Silbershatz H, Kannel WB (1998). Prediction of coronary heart disease using risk factor categories. Circulation..

[33] Fowkes FG, Murray GD, Butcher I, Heald CL, Lee RJ, Chambless LE, Folsom AR, Hirsch AT, Dramaix M (2008). Ankle brachial index combined with Framingham risk score to predict cardiovascular events and mortality: a meta-analysis. JAMA..

[34] Horne R, Weinman J (1999). Patients' beliefs about prescribed medicines and their role in adherence to treatment in chronic physical illness. J Psychosom Res.

[35] Horne R, Weinman J, Hankins M (1999). The beliefs about medicines questionnaire: the development and evaluation of a new method for assessing the cognitive representation of medication. Psychol Health.

[36] Wangdahl J, Lytsy P, Martensson L, Westerling R (2014). Health literacy among refugees in Sweden - a cross-sectional study. BMC Public Health.

[37] Herdman M, Gudex C, Lloyd A, Janssen M, Kind P, Parkin D, Bonsel G, Badia X (2011). Development and preliminary testing of the new five-level version of EQ-5D (EQ-5D-5L). Qual Life Res.

[38] Janssen MF, Pickard AS, Golicki D, Gudex C, Niewada M, Scalone L, Swinburn P, Busschbach J (2013). Measurement properties of the EQ-5D-5L compared to the EQ-5D-3L across eight patient groups: a multi-country study. Qual Life Res.

[39] Nordanstig J, Wann-Hansson C, Karlsson J, Lundstrom M, Pettersson M, Morgan MB (2014). Vascular quality of life Questionnaire-6 facilitates health-related quality of life assessment in peripheral arterial disease. J Vasc Surg.

[40] Devlin NJ, Brooks R (2017). EQ-5D and the EuroQol group: past, present and future. Appl Health Econ Health Policy.

[41] Kumlien C, Nordanstig J, Lundstrom M, Pettersson M (2017). Validity and test retest reliability of the vascular quality of life Questionnaire-6: a short form of a disease-specific health-related quality of life instrument for patients with peripheral arterial disease. Health Qual Life Outcomes.

[42] Schwarzer R, Jerusalem M, Weinman J, Wright S, Johnston M (1995). Generalized self-efficacy scale, causal and control beliefs. Measures in health psychology: a user’s portfolio.

[43] Love J, Moore CD, Hensing G (2012). Validation of the Swedish translation of the general self-efficacy scale. Qual Life Res.

[44] Wilde B, Larsson G, Larsson M, Starrin B (1994). Quality of care. Development of a patient-centred questionnaire based on a grounded theory model. Scand J Caring Sci.

[45] Wilde Larsson B, Larsson G (2002). Development of a short form of the quality from the Patient's perspective (QPP) questionnaire. J Clin Nurs.

[46] Schulz KF, Altman DG, Moher D, Group C (2010). CONSORT 2010 statement: updated guidelines for reporting parallel group randomised trials. BMJ..

[47] Chan AW, Tetzlaff JM, Altman DG, Laupacis A, Gotzsche PC, Krleza-Jeric K, Hrobjartsson A, Mann H, Dickersin K, Berlin JA (2013). SPIRIT 2013 statement: defining standard protocol items for clinical trials. Ann Intern Med.

[48] Rich MW, Chyun DA, Skolnick AH, Alexander KP, Forman DE, Kitzman DW, Maurer MS, McClurken JB, Resnick BM, Shen WK (2016). Knowledge gaps in cardiovascular Care of Older Adults: a scientific statement from the American Heart Association, American College of Cardiology, and American Geriatrics Society: executive summary. J Am Geriatr Soc.

[49] Rolstad S, Adler J, Ryden A (2011). Response burden and questionnaire length: is shorter better? A review and meta-analysis. Value Health.

[50] Moore GF, Audrey S, Barker M, Bond L, Bonell C, Hardeman W, Moore L, O'Cathain A, Tinati T, Wight D (2015). Process evaluation of complex interventions: Medical Research Council guidance. BMJ..

[51] World Medical Association (2013). World medical association declaration of Helsinki: ethical principles for medical research involving human subjects. JAMA..

